# Effect of acupuncture on vascular cognitive impairment (VCI): A randomized controlled trial

**DOI:** 10.1097/MD.0000000000044257

**Published:** 2025-09-19

**Authors:** Lin Bai, Hong-Liang Cheng, Wen-Dong Zhang, Pei-Jia Hu, Fang-Yuan Xu, Ying-Quan Liu, Fan Dai, Li-Yao Zhang

**Affiliations:** a The Second Affiliated Hospital of Anhui University of Chinese Medicine, Hefei, China; b Anhui University of Chinese Medicine, Hefei, China.

**Keywords:** acupuncture, neuroinflammation, post-stroke, randomized controlled trial, vascular cognitive impairment (VCI)

## Abstract

**Background:**

Vascular cognitive impairment (VCI) is a condition associated with cerebrovascular diseases, which causes a heavy burden on both individuals and society. Acupuncture has been used extensively in China to treat these complications. However, the therapeutic efficacy of this treatment remains uncertain. Consequently, we aimed to investigate the clinical effects of acupuncture on VCI.

**Methods:**

Patients (n = 97) were randomly divided into the intervention (n = 48) and the control (n = 49) groups. The intervention group was given donepezil hydrochloride orally once a day for 4 weeks, and the intervention group was combined with acupuncture treatment on the basis of control group once daily, 6 days a week, for a total of 4 weeks. Mini-Mental State Examination (MMSE) and Montreal Cognitive Assessment scores were performed before the intervention and after the intervention (4 weeks post-intervention). The levels of interleukin (IL)-1β and IL-6 were measured before the intervention and after the intervention (4 weeks post-intervention). Finally, the clinical effective rate was calculated according to the MMSE scores before and after intervention.

**Results:**

Following the intervention, significant differences were observed between the intervention and control groups. After 4 weeks, MMSE and Montreal Cognitive Assessment scores were significantly increased (*P* < .001), and IL-1β and IL-6 levels were significantly decreased (*P* < .001).

**Conclusion:**

Acupuncture treatment can improve the cognitive function of patients with VCI and decrease the levels of IL-1β and IL-6. These findings strongly support the efficacy of acupuncture as a therapeutic intervention in patients with VCI.

## 1. Introduction

Cognitive impairment is a complex neurocognitive disorder that can occur secondary to various cerebrovascular diseases. Patients with cognitive impairment exhibit cognitive, speech, memory, emotion, and other disorders as the main symptoms, which leads to serious decline in self-care ability, loss of social function, reduction in their quality of life, and damage to their physical and mental health.^[1]^ Currently, there are approximately 55 million cases of cognitive impairment worldwide, which is projected to quadruple by 2050.^[2]^ In China, the number of patients with cognitive impairment was 15.33 million in 2019, and it is expected that by 2050, the number of patients with cognitive impairment will rise to 45.33 million.^[3]^ The prevalence of cognitive impairment increases with age,^[4]^ thereby placing a heavy burden on individuals and the society.^[5]^ Among the types of cognitive impairment, vascular cognitive impairment (VCI) is the second leading cause of cognitive impairment in older adults, second only to Alzheimer disease.^[6]^ A recent study showed that the prevalence of VCI among 10-year stroke survivors was 61%.^[7]^ However, VCI has preventive and therapeutic potential and is a popular subject in the field of cognitive research. Therefore, identifying new targets for VCI treatment is of great theoretical and clinical significance.

Acupuncture has a long history of use for the treatment of cognitive disorders. More than 2000 years ago, Huangdi Neijing (The Yellow Emperor’s Inner Canon) documented the application of acupuncture in the treatment of dementia. Modern studies have shown that acupuncture can be used as a safe and effective alternative and complementary treatment for cognitive disorders, and this has also been recognized by the World Health Organization.^[8]^ Previous studies have shown that acupuncture can inhibit neuroinflammation, stimulate the vagus nerve,^[9]^ have anti-apoptotic effects on neurons, improve synaptic plasticity, and regulate brain energy metabolism^[10]^ to improve abstract reasoning, memory, and orientation.^[11]^ Inflammatory injury is regarded as a key factor in the pathogenesis of VCI. Studies have found that acupuncture can alleviate inflammation-related cognitive impairment by inhibiting the nuclear factor-kappa B signaling pathway^[12]^ and can also reverse the upregulation of inflammatory gene expression in VCI models, thereby improving the state of cognitive impairment.^[13]^ Studies have also shown that acupuncture can effectively alleviate vascular cognitive dysfunction and reduce apoptosis, inflammation, and autophagy by regulating the autophagy–inflammation pathway mediated by mTOR/NLRP3.^[14]^

In China, acupuncture is widely used to treat patients with cognitive impairment after stroke. Therefore, we aimed to investigate the efficacy of acupuncture in the treatment of VCI after stroke.

## 2. Methods

### 2.1. Ethical approval of research protocol

This assessor-participant-blinded, single-center, randomized controlled trial was conducted on patients diagnosed with VCI. The study was conducted between January 15, 2022, and May 26, 2024, at the Second Affiliated Hospital of Anhui University of Chinese Medicine and was approved by the Ethics Committee of the Second Affiliated Hospital of the Anhui University of Traditional Chinese Medicine (approval number: 2022-zj-40). To maintain the integrity of the randomized controlled trial, a skilled clinical trial specialist was enlisted to supervise the process, offer expert recommendations, and implement the necessary modifications to resolve project-related challenges.^[15]^

### 2.2. Participants

The study included outpatients and inpatients who received treatment at the hospital between January 15, 2022, and May 26, 2024. All eligible patients provided informed consent before participation. In addition, all participants satisfied the diagnostic criteria for VCI.^[16]^ The baseline demographic and clinical characteristics of the trial groups are presented in Table 1. The male-to-female ratio in the intervention group 48 was 21:27, whereas that in the control group 49 it was 23:26. All patients met the diagnostic criteria for ischemic stroke and cognitive impairment.

**Table 1:** Clinical characteristics of patients in the intervention group and the control group.

Variable	Intervention group (n = 48)	Control group (n = 49)	*t*/*X*^2^-values	*P*-value
Sex (male/female)	21/27	23/26	0.099	.554
Age (yr)	67.08 ± 4.59	63.27 ± 5.09	3.876	.350
Course of disease (mo)	6.31 ± 3.40	4.65 ± 2.53	2.730	.143
Hypertension	12 (25.0%)	15 (30.6%)	0.380	.387
Diabetes	23 (47.9%)	21 (42.9%)	0.250	.224

The inclusion criteria were as follows: age of 50 to 80 years; a dominant stroke event and disease course within 90 days; no history of transient ischemic attack; Mini-Mental State Examination (MMSE) scores ranging from 16 to 26 points; Montreal Cognitive Assessment (MoCA) scores ranging from 19 to 25; the patient was conscious, generally in good condition, and could cooperate with examination and treatment; the patient enrolled voluntarily and signed the treatment consent form; and the length of education was at least 7 years.

The exclusion criteria were as follows: age < 50 years or > 80 years; cognitive impairment caused by nonvascular factors; no correlation between cognitive impairment and brain impairment; MMSE ≤ 15 or MoCA ≤ 19; severe damage to other organs; and inability to complete cognitive tests owing to motor, sensory, or language impairments.

If serious adverse reactions occurred, the clinical trial was terminated.

### 2.3. Study procedures

**Figure 1.**
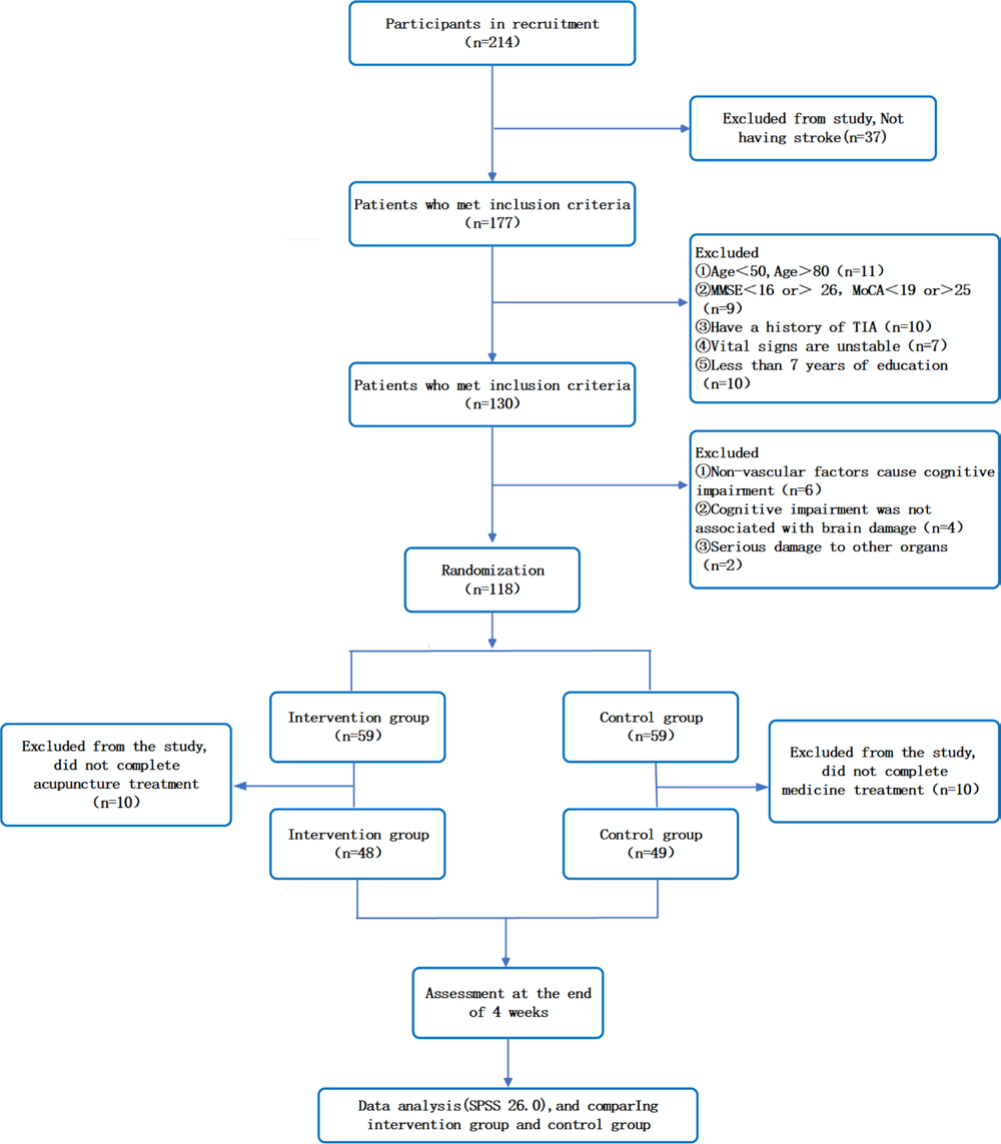
Flow chart of the trial.

The study population included 214 patients with VCI who were recruited at the study site (Fig. 1). A total of 118 participants were initially enrolled, and because of the challenges encountered during the recruitment process, only 97 patients met the eligibility criteria within the specified timeframe. A numbered randomization list was generated using Microsoft Excel (Hefei, Anhui Province, China), and patients were subsequently ordered according to their enrollment time. Random numbers were produced using the random number table method, and patients meeting the inclusion criteria were randomly assigned to either the intervention or control group. All healthcare personnel, except acupuncturists, were blinded to the group assignments before the enrollment of each participant.

All adverse events that occurred during treatment were systematically recorded in case report forms. These events included syncope during acupuncture sessions, infections, needlestick injuries, and other complications.

### 2.4. Treatment methods

The patients received standard inpatient and outpatient care according to hospital protocols. Furthermore, individuals in the intervention group were treated with acupuncture administered by a highly experienced practitioner who had 15 years of clinical experience and held certification from the China Association of Acupuncture and Moxibustion. During this period, none of the patients participated in any rehabilitation treatment other than acupuncture.

#### 2.4.1. Control group

Donepezil hydrochloride tablets were administered orally once a day for 4 weeks (specification: 5 mg per tablet; manufacturer: Jiangsu Hausen Pharmaceutical Group Co., Ltd; approval number: H20030472).

#### 2.4.2. Intervention group

In addition to the oral donepezil hydrochloride tablets, the intervention group was treated with “Tongdu Tiaoshen” acupuncture. The key points used for all patients in the intervention (acupuncture) group were DU20 (Baihui), DU24 (Shenting), DU16 (Fengfu), DU14 (Dazhui), DU9 (Zhiyang), DU4 (Mingmen), and DU3 (Yaoyangguan). After routine disinfection of the acupoints, the patients were placed in the prone position. When selecting the DU20 point, the acupuncturist used 0.38 × 25 mm acupuncture needles to stab flat backwards with a depth of 20 mm. The flat acupuncture depth of the DU24 acupoint was 12.5 mm with 0.38 × 25 mm needles. For DU16, 0.38 × 40 mm acupuncture needles were slowly inserted 25 mm into the mandibular direction. For DU14, DU9, DU4, and DU3, the direct injection method was used, with 0.38 × 50 mm needles and the injection depth of 25 to 40 mm. All the needles were incubated for 40 minutes. The intervention treatment was administered to the patients 6 times per week for 4 weeks.

### 2.5. Outcome measures

#### 2.5.1. Main outcome indicators

MMSE^[17]^: it is a simple scale used to assess an individual’s cognitive function. The MMSE consists of 19 tests, including: time cognition, place cognition, memory, attention, numeracy, and comprehension abilities, with a perfect score of 30 and a score below 27 indicating cognitive impairment.

MoCA^[18]^: the MoCA consists of 11 tests, including 8 cognitive areas, including attention and concentration ability, executive ability, memory ability, language ability, visual structure skills, abstract thinking ability, calculation ability, and orientation ability. The total MoCA score is 30, and a score < 26 indicates cognitive impairment. MoCA is highly sensitive and can be used for rapid screening of mild cognitive impairment.

#### 2.5.2. Secondary outcome indicators

##### 2.5.2.1. Inflammation

Before and after treatment, 5 mL of venous blood was collected from patients on a fasting diet and centrifuged at a rotating speed of 3500 rpm for 8 minutes. Serum was separated, and serum interleukin (IL)-1β and IL-6 were detected before and after treatment using double-antibody sandwich enzyme-linked immunosorbent assay.

##### 2.5.2.2. Efficacy evaluation

Evaluation of cognitive function efficacy was based on the criteria for diagnosis, differentiation, and efficacy of vascular dementia,^[19]^ and efficacy was evaluated according to the MMSE scale: clinical response rate = (MMSE score after treatment − MMSE score before treatment)/MMSE score before treatment × 100%. Efficacy index ≥ 85% was considered very strong progress, ≥50% and <85% was considered strong progress, ≥20% and <50% was considered progress, and <20% was considered invalid.

### 2.6. Data analysis

Data were analyzed using Statistical Package for Social Sciences (SPSS) software (version 26; SPSS Inc., Chicago). Quantitative variables are presented as the mean ± standard deviation. The Shapiro–Wilk test was used to assess the normality of the distribution of variables. For variables demonstrating a normal distribution, intragroup comparisons were performed using a paired-sample *t* test, whereas intergroup comparisons were performed using an independent-samples *t* test. In instances where the distribution was non-normal, the Mann–Whitney *U* test was used to compare the clinical features between the 2 groups. Qualitative variables are presented as numbers (%).

## 3. Results

### 3.1. Baseline characteristics

The comparison of the 2 groups is presented in Table 1. No obvious differences in sex, age, course of disease, hypertension, and diabetes were observed between the 2 groups (*P* > .05).

### 3.2. Assessment of MMSE and MoCA scores

Following the intervention, participants in the intervention group exhibited significantly greater increases in MMSE and MoCA scores than those in the control group (*P* < .01), as shown in Table 2. This indicates that “Tongdu-Tiaoshen” acupuncture improves cognitive function in patients.

**Table 2:** Comparison of MMSE and MoCA scores between the intervention group and the control group.

Groups	Number of cases	MMSE scores		MoCA scores	
		Before intervention	After intervention	Before intervention	After intervention
Intervention group	48	20.29 ± 2.43	24.90 ± 3.24	21.02 ± 1.86	25.85 ± 1.95
Control group	49	21.90 ± 2.09	22.65 ± 2.78	20.51 ± 1.47	23.49 ± 2.08
*t*-values		-3.488	4.002	1.499	5.209
*P*-value		.226	.000	.079	.000

MMSE = Mini-Mental State Examination, MoCA = Montreal Cognitive Assessment.

### 3.3. Assessment of IL-1β and IL-6 levels

No significant between-group differences in IL-1β or IL-6 levels were observed before the intervention (*P* > .05). After treatment, the IL-1β and IL-6 levels decreased in both the groups. The decrease in the intervention group was more obvious than that in the control group (*P* < .01), as shown in Table 3. This indicates that ‘Tongdu Tiaoshen’ acupuncture decreases IL-1β and IL-6 levels.

**Table 3:** Comparison of IL-1β and IL-6 levels between the intervention group and the control group.

Groups	Number of cases	IL-1β (ng/mL)		IL-6 (pg/mL)	
		Before intervention	After intervention	Before intervention	After intervention
Intervention group	48	91.12 ± 8.18	73.88 ± 7.78	13.54 ± 1.97	5.83 ± 2.21
Control group	49	88.04 ± 9.76	82.54 ± 9.07	12.88 ± 2.20	7.92 ± 1.92
*t*-values		1.682	4.504	1.565	-4.855
*P*-value		.364	.000	.176	.000

IL = interleukin.

### 3.4. Clinical efficacy assessment

The clinical effect in the intervention group was greater than that in the control group; however, the difference was not statistically significant (*P* > .05). The total efficiencies of the intervention and control groups were 89.58% and 75.51%, respectively. The differences in efficacy rates between the 2 groups were statistically significant (*P* < .05), as shown in Table 4.

**Table 4:** Comparison of clinical efficacy between the intervention group and the control group.

Groups	Very strong progress	Strong progress	Progress	Invalid	Total effective rate
Intervention group (n = 48)	17 (35.42%)	13 (27.08%)	13 (27.08%)	5 (10.42%)	43 (89.58%)
Control group (n = 49)	7 (14.29%)	10 (20.41%)	20 (40.82%)	12 (24.49%)	37 (75.51%)
χ^2^-values					8.916
*P*-value					.030

## 4. Discussion

VCI is a broad concept that includes cognitive impairments caused by ischemic and hemorrhagic cerebrovascular diseases.^[20]^ In terms of severity, VCI can range from mild VCI to vascular dementia.^[21]^ Many cardiovascular disease risk factors such as hypertension, diabetes, smoking, atrial fibrillation, dyslipidemia, and physical inactivity increase the risk of VCI. Moreover, this risk increases in dose-dependent manner with age^[22]^ and was found to predict the risk of cognitive impairment at 20^[23]^ and even 40 years later.^[24]^ However, except for age, risk factors for VCI are preventable and require multimodal interventions, including diet, exercise, cognitive training, vascular risk monitoring, and lifestyle changes.^[25]^

Chinese medicine has a long history of use for the treatment of cognitive impairment. As early as Huangdi Neijing (The Yellow Emperor’s Inner Canon), there is a record of cognitive impairment, which suggests that cognitive impairment is located in the brain and is most closely related to the Du Meridian. “Tongdu Tiaoshen” acupuncture is a special acupuncture therapy based on the holistic concept of traditional Chinese medicine, which has been applied in our hospital for > 30 years, and the curative effect is remarkable. This theory holds that the first choice for treating stroke and related diseases should be the acupoint of Du Meridian. There are 3 main reasons: first, the Du Meridian has the function of leading the whole body Yang Qi and regulating the Qi and blood of Yang meridians. Second, in terms of Du Meridian circulation and anatomical structure, the Du Meridian runs through the kidney, spinal cord, and brain and is the bridge between the kidney and brain. According to Chinese medicine, the kidney is the source of life and contains the essence of reproduction. The essence of reproduction can be converted into the marrow and then passed through the spinal cord to brain. Third, a branch of the Du Meridian runs through the heart. According to traditional Chinese medicine, the heart is in charge of the mind and cognition and dominates all life activities of the human body. The blood of the heart reaches the brain through the Du Meridian, thereby improving the cognition of the brain. Based on this theory, acupuncture treatment was designed for this clinical trial. The acupoints that we selected belonged to the acupoints of the Du Meridian, and we used the MMSE and MoCA scores to evaluate treatment efficacy. After treatment, we found that the MMSE and MoCA scores in the acupuncture group significantly increased, indicating that “Tongdu Tiaoshen” acupuncture improves cognitive function in patients. The clinical efficacy rate of the acupuncture group was 89.58%, which was much higher than that of the control group. These findings indicate that acupuncture is a simple and effective technique that is suitable for extensive clinical application in VCI.

There are many mechanisms of cognitive impairment, such as oxidative stress,^[26]^ inflammation,^[27]^ blood–brain barrier dysfunction,^[28]^ dietary patterns,^[29]^ and intestinal flora dysbiosis.^[30]^ Neuroinflammation is an immune response triggered by central nervous system glial cells and is considered to play a key role in the mechanism of cognitive impairment.^[31]^ It can result in synaptic damage, neuronal dysfunction, cell death, and impaired neurogenesis.^[32]^ Chronic cerebral hypoperfusion (CCH)-induced ischemia and hypoxia can trigger neuroinflammation, which significantly contributes to the onset and progression of VCI.^[33]^

Inflammatory injury is a key factor in the pathogenesis of VCI. Primary hemodynamic damage, vascular changes, and functional disorders after stroke can lead to VCI. Vascular changes can affect innate immunity and activate white blood cells and antigen-presenting cells. Damaged vascular tissues produce adenosine triphosphate and increase inflammatory cytokine levels. Continuous aggravation of vascular inflammation damages the brain tissue and can alter inflammatory processes throughout the body. These immune responses are closely related to diseases such as hypertension, diabetes, hyperlipidemia, and arteriosclerosis, which are risk factors for VCI.^[34]^ These risk factors induce vascular inflammation, which clinically manifests as inflammation and circulatory disorders. Pathologically, neuronal damage, changes in the shape and tissue of blood vessels, and induction of vascular fibrosis can be observed, indicating activation of the immune response.^[35]^

VCI occurs in the cerebral hypoperfusion area. CCH occurs throughout the VCI process and is the primary cause of VCI. CCH activates the inflammatory molecular and cellular damage cascade, leading to the destruction of the blood–brain barrier and neurodegeneration, thereby regulating the microenvironment within the brain parenchyma.^[36]^ CCH can directly activate inflammasome signaling pathways (including NLRP3 and AIM2 inflammasome) and increase the levels of inflammatory factors, such as IL-1β, IL-6, IL-8, and C-reactive protein.^[37]^

Studies have shown that pro-inflammatory cytokines such as IL-1β and IL-6 maintain a chronic neuroinflammatory state, manifested by cognitive impairment.^[38]^ The levels of inflammatory biomarkers in plasma and cerebrospinal fluid of patients with VCI are changed, indicating that neuroinflammation is involved in the pathogenesis of VCI.^[39]^ In this study, the levels of IL-1β and IL-6 decreased in the acupuncture group after treatment, indicating that acupuncture regulates neuroinflammation by alleviating cerebral ischemia and hypoxia and activating immune cells.

This study has several limitations. First, the duration of acupuncture treatment was limited to 4 weeks. This brief treatment period may have influenced the recovery of patients’ cognitive function. Second, the study was constrained by the small sample size; although 214 patients were initially recruited, only 97 completed the trial. Future research should aim to increase the sample size of the validation studies to yield robust results. Third, although we assessed the efficacy of acupuncture across the study groups, we did not consider the site of stroke onset, which may have influenced the outcome evaluation. Fourth, there were only donepezil and donepezil + acupuncture groups in the grouping and no placebo group. This may have led to insufficient persuasiveness in acupuncture treatment for VCI. Fifth, the dosage of donepezil hydrochloride was 5 mg for 1 month; therefore, it may not have been effective. In the future, we intend to incorporate objective criteria and systematically document the locations of cerebrovascular blockages to ascertain whether the impact of acupuncture on VCI is associated with the site of vascular obstruction. Meanwhile, we will add a placebo group, prolong the treatment interval, increase the dosage of donepezil tablets, and make the trial rigorous.

## 5. Conclusion

“Tongdu Tiaoshen” acupuncture for VCI is effective and safe. It can effectively improve the cognitive ability of patients and decrease the levels of IL-1 and IL-6 by ameliorating cerebral ischemia and hypoxia and inhibiting neuroinflammation. Thus, “Tongdu Tiaoshen” acupuncture is a therapeutic modality that can be integrated into clinical practice.

## Acknowledgments

We would like to thank all the participants of this study. IL-1β and IL-6 were measured at the clinical laboratory of the Second Affiliated Hospital of Anhui University of Chinese Medicine.

## Author contributions

**Data curation:** Fang-Yuan Xu.

**Formal analysis:** Ying-Quan Liu.

**Methodology:** Wen-Dong Zhang.

**Project administration:** Hong-Liang Cheng.

**Software:** Fan Dai, Li-Yao Zhang.

**Visualization:** Fan Dai, Li-Yao Zhang.

**Writing – original draft:** Lin Bai.

**Writing – review & editing:** Pei-Jia Hu.
